# Sepsis and AKI in ICU Patients: The Role of Plasma Biomarkers 

**DOI:** 10.1155/2012/856401

**Published:** 2012-02-14

**Authors:** Paolo Lentini, Massimo de Cal, Anna Clementi, Angela D'Angelo, Claudio Ronco

**Affiliations:** ^1^Department of Nephrology, San Bassiano Hospital, 36061 Bassano del Grappa, Italy; ^2^Division of Nephrology, University of Padua, 35100 Padua, Italy; ^3^Department of Nephrology, San Bortolo Hospital, 36100 Vicenza, Italy

## Abstract

Given the higher mortality rate of ICU patients with sepsis and AKI, we decided to investigate the possible correlation between serum biomarkers of organ damage, and endotoxin activity in ICU septic patients. Ninety-eight consecutive adult patients were enrolled in this study. Patients were divided in two groups depending on the presence of sepsis. Fifty-six patients had sepsis, while forty-two patients were nonseptic. Among septic patients, twenty-four subjects developed AKI, while thirty-two did not. AKI occurred in fourteen patients without sepsis as well. The levels of NGAL, BNP, and AOPP were significantly higher among septic patients compared with nonseptic subjects (*P* < 0.001). Among septic patients, subjects who developed AKI showed significant higher levels of NGAL and AOPP (*P* = 0.0425) and BNP (*P* = 0.0327). Among patients who developed AKI, a significant difference was found only in terms of AOPP levels between septic and nonseptic patients. The correlation between endotoxin activity and BNP in septic patients and the increase in the levels of NGAL, BNP, and AOPP in case of sepsis and AKI, in particular if they are associated, indicate a multiorgan involvement in these two conditions.

## 1. Introduction

Sepsis, defined as a systemic inflammatory response syndrome (SIRS) associated with an infectious disease [1, 2], is a primary cause of morbidity and mortality in ICU [3] and critically ill patients. Mortality rates range from 20% for sepsis, to 40% for severe sepsis, to 60% for septic shock in ICU patients [4].

Gram-negative bacteria are implicated in 50–60% of sepsis, with Gram-positive bacteria accounting for a further 35–40% of cases. The remainder of causes are due to the less common causes of fungi, viruses, and protozoa [5].

The heat-stable toxic component of Gram-negative bacteria, identified for the first time by Pfeiffer at the end of the 19th century [6, 7] and called “endotoxin”, is considered to play an important role in the pathogenesis of septic shock [8]. It causes the release of different cytokines, such as interleukin-1 (IL-1) and tumor necrosis factor-*α* (TNF-*α*), and interacts with the complement pathway and the coagulation system [8, 9].

Sepsis is also a contributing factor in more than 20% of cases of acute kidney injury (AKI) in ICU patients, with cases severe enough to require renal replacement therapy [10–12]. AKI occurs in 35–65% of ICU admissions, and most studies show a threefold to fivefold increase in the risk of death among patients with AKI compared to patients without AKI.

Given the higher mortality rate of ICU patients with sepsis and AKI, we decided to investigate the possible correlation between serum biochemical markers of organ damage, such as neutrophil gelatinase-associated lipocalin (NGAL), advanced oxidation protein products (AOPP), and brain natriuretic peptide (BNP) and endotoxin activity in ICU septic patients. Moreover, comparisons of the levels of these biomarkers were made between septic and nonseptic patients, septic patients with or without AKI, and between patients who developed AKI with or without sepsis.

## 2. Material and Methods

### 2.1. Study Population

Ninety-eight consecutive adult patients, admitted to ICU of San Bortolo Hospital, Vicenza, Italy, between October 2008 and august 2010, were enrolled in this study. Patients were divided in two groups depending on the presence of sepsis, defined as systemic inflammatory response syndrome (SIRS) associated with an infectious process. SIRS was considered to be present when at least two of the following criteria were present: temperature above 38°C or below 36°C, heart rate above 90 beats/min, respiratory rate above 20 breaths/min or partial pressure of carbon dioxide below 32 mmHg, and white blood cell count above 12,000 mm^3^ or below 4,000 mm^3^. Fifty-six patients had sepsis, while forty-two patients were nonseptic. Clinical and biochemical characteristic of septic patients are summarized in Table 1.

Among septic patients, twenty-four subjects developed AKI, defined by RIFLE criteria, while thirty-two did not. AKI occurred in fourteen patients without sepsis as well.

Within four hours after admission blood samples were taken for EAA (endotoxin activity assay), NGAL, and BNP measurement. EDTA was used as an anticoagulant. Heparinized blood samples were collected for AOPP evaluation.

Correlation between NGAL, AOPP, BNP and endotoxin activity in septic patients was evaluated. Moreover, comparisons of the levels of these biomarkers were made between septic and non septic patients, septic patients with or without AKI, and between patients who developed AKI with or without sepsis.

### 2.2. Endotoxin Activity Assay (EAA)

Serum endotoxin activity was measured by the EAA^tm^ which measures the degree of chemiluminescence of the circulating neutrophil population induced by the exposure to endotoxin.

The test is based on the interaction between the endotoxin and a specific antiendotoxin antibody. Complement components opsonize the endotoxin-antibody complex. The opsonized immune complex primes neutrophils in the blood to enhance their respiratory burst in response to zymosan. The respiratory burst of the neutrophils yields oxidants that react with luminal in the reaction mixture to emit chemiluminescence.

The chemiluminescence can then be detected in a photon-counting luminometer (SmartLine TL, Berthold Detection Systems, Pforzheim, Germany).

A basal activity measurement (Tube 1) in the absence of the specific antiendotoxin antibody measures the nonspecific oxidative burst of the patient's neutrophils. An additional control measurement including the specific antiendotoxin antibody and an excess of exogenous endotoxin (Tube 3) measures the maximum oxidative burst of the patient's neutrophils. The test measurement (Tube 2) includes the specific antibody to measure the neat level of endotoxin activity. The EAA^tm^ level is calculated by normalizing the chemiluminescence in the test sample (Tube 2) against the maximum chemiluminescence (Tube 3), correcting both measurements for the basal activity chemiluminescence (Tube 1).

Endotoxin activity levels are expressed as units on a scale ranging from 0 to 1 

0.00–0.39: EAA^tm^ units: low endotoxin activity level,
0.40–0.59: EAA^tm^ units: intermediate endotoxin activity level,
≥0.60: EAA^tm^ units: high endotoxin activity level.

### 2.3. NGAL and BNP Measurement

Plasma samples for NGAL and BNP measurement were stored at minus 80 degrees Celsius to be analyzed subsequently. Plasma NGAL and BNP were measured with fluorescence-based immunoassay with the Triage point-of-care analyzer (Biosite Inc., San Diego, CA, USA), which allows a rapid quantitative measurement of NGAL and BNP concentration in EDTA-anticoagulated whole blood or plasma. NGAL and BNP concentrations were expressed as nanograms per millilitre (ng/mL) and pictograms per millilitre (pg/mL), respectively.

### 2.4. AOPP Measurement

AOPP levels were measured by spectrophotometry and calibrated with Chloramine-T solutions (Sigma Chemical Co., St. Louis, MO, USA), which adsorb at 340 nm in presence of potassium iodide. Two hundred microliters of plasma diluted 1/5 in PBS, and 20 *μ*L of acetic acid were mixed and calibrated versus the standard reference of 200 *μ*L Chloramine-T solution (0–100 *μ*mol/L) with 20 *μ*L of acetic acid and 10 *μ*L of potassium iodide.

The absorbance of the reaction mixture was read at 340 nm against a blank containing 200 *μ*L of PBS, 10 *μ*L of potassium iodide, and 20 *μ*L of acetic acid. AOPP concentrations were expressed as micromoles per liter of chloramine-T equivalents (*μ*mol/L).

### 2.5. Statistical Analysis

Statistical analysis was performed with the use of SPSS software version 15.0. Categorical variables were expressed as percentages; continuous variables were expressed as means ± standard deviation (parametric variables) or median (interquartile range; nonparametric variable). Differences between groups were analyzed using Student *t*-test and Mann-Whitney test as appropriate. Correlation was performed with the use of the Spearman rank coefficient. Two-tailed probability values of <0.05 were considered statistically significant.

## 3. Results

Septic patients were divided in three groups depending on EAA levels. EAA < 40: 8 patients; EAA 40–60: 17 patients; EAA > 60: 31 patients.

**Figure 1:**
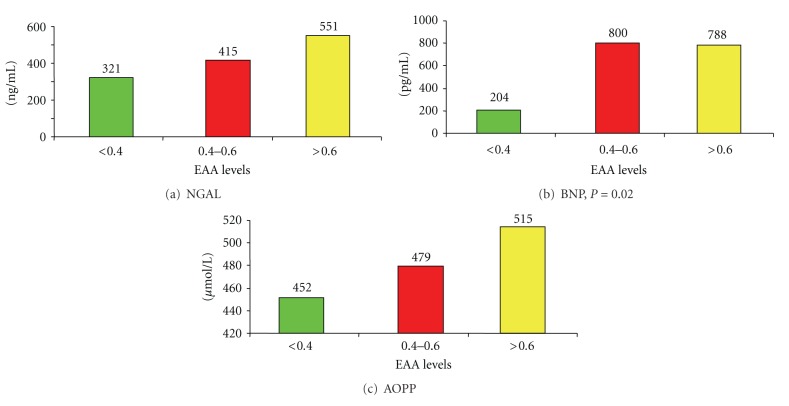
Correlation between EAA (<0.40; 0.40–0.60; >0.60 units) and the levels of NGAL, BNP, and AOPP.

A significant correlation (*P* = 0.02) was found only between endotoxin activity and BNP levels of septic patients (Figure 1). The levels of NGAL, BNP, and AOPP were significantly higher among septic patients compared with nonseptic subjects (*P* < 0.001) (Table 2). Among septic patients, subjects who developed AKI showed significant higher levels of NGAL and AOPP (*P* = 0.0425) and BNP (*P* = 0.0327) (Table 3). Among patients who developed AKI, a significant difference was found only in terms of AOPP levels between septic and non septic patients (Table 4).

## 4. Discussion

As reported by Marshall et al., intermediate and high levels of endotoxin activity are often found in ICU septic patients [13], and they seem to correlate with the severity of the disease, in particular with the hemodynamic alterations [14]. Sepsis, indeed, frequently causes cardiac abnormalities and kidney dysfunction [15, 16] and, for this reason, can be considered as an important cause of type 5 cardiorenal syndrome [17].

In this study we investigated the possible correlation between endotoxin activity in septic ICU patients and biochemical markers of organ damage, such as NGAL, AOPP, and BNP.

As shown in Figure 1, a significant correlation was found only between endotoxin activity and BNP levels of septic patients (*P* = 0.02). BNP is considered to be a good diagnostic and prognostic biomarker, especially among patients with congestive heart failure [18]. Elevated levels of BNP are independent predictors of cardiovascular morbidity and mortality, both in patients with normal and impaired renal function, thus emphasizing the value of BNP in the assessment of cardiorenal syndrome [19]. In our study, intermediate and higher levels of endotoxin activity, which predict an elevated risk for developing severe sepsis, were associated with higher levels of BNP, which result from cardiac dysfunction induced by sepsis.

We also compared the levels of NGAL, AOPP, and BNP between septic and non septic patients, septic patients with or without AKI, and between patients who developed AKI with or without sepsis.

Serum NGAL has been shown to increase before serum creatinine in case of acute kidney injury [20] and has therefore become a novel early biomarker of acute renal damage [21]. Moreover, it was found to rise in patients with congestive heart failure, thus indicating a link between cardiac dysfunction and renal injury [18, 22, 23].

Critically ill patients also present increased levels of AOPP, induced by the overproduction of reactive oxygen species (ROS) and the subsequent depletion of the antioxidant endogenous stores. AOPP levels were demonstrated to correlate with the risk to develop severe sepsis and with the severity of AKI in ICU patients [19, 24].

In our study, the levels of NGAL, BNP, and AOPP were significantly higher among septic patients compared with non septic subjects (*P* < 0.001), as shown in Table 2. Among septic patients, subjects who developed AKI showed significant higher levels of NGAL and AOPP (*P* = 0.0425) and BNP (*P* = 0.0327) (Table 3). Among patients who developed AKI, a significant difference was found only in terms of AOPP levels between septic and non septic patients (Table 4).

These data suggest that sepsis and AKI are responsible for the increase in the level of the three biomarkers, in particular if they are associated. When limiting to the AKI patients, there was no significant difference in terms of NGAL and BNP levels between septic and non septic patients. The reason for this finding remains to be clarified. A possible explanation could be that renal damage alone can cause a similar increase in the level of the two biomarkers, independently on the presence of sepsis.

## 5. Conclusions

In septic ICU patients endotoxin activity correlates with BNP levels. NGAL, AOPP, and BNP levels seem to be higher in patients with sepsis and AKI, in particular if they are associated. In case of AKI, a significant difference between septic and nonseptic patients was found only for AOPP levels.

NGAL, AOPP, and BNP increase in case of sepsis, thus indicating both cardiac and renal impairment. For this reason, the rise in their levels in this condition can allow clinicians to individualize patients at higher risk for developing severe sepsis and therefore at higher risk of death.

## Figures and Tables

**Table 1:** Clinical and biochemical characteristics of septic patients.

	Septic patients (*N* = 56)
Male sex (%)	33.9
Age (years)	69 (48.7 to 74.2)
Serum creatinine (mg/dL)	1.64 (1.04 to 2.97)
Temperature (°Celsius)	36.4 ± 2.0
WBC (million cells/mcL)	12.3 ± 9.4
Platelets (10^3^/*μ*L)	144.6 ± 110.9
pH	7.359 ± 0.154
Na (mmol/L)	139.5 ± 6.1
K (mmol/L)	4.2 ± 1.1
PaO_2_/FiO_2_ (mmHg)	206.4 ± 112.5
Sofa score	10 (8 to 12)
Died (%)	32.1

WBC: white blood cells; SOFA score: sequential organ failure assessment.

**Table 2:** Comparison of biochemical markers between septic patients and nonseptic patients.

	Septic pts (*N* = 56)	Nonseptic pts (*N* = 42)	*P* value
Male sex (%)	33.9	66.7	0.0013
Age (years)	69 (48.7 to 74.2)	67 (59 to 75)	0.83
Creatinine (mg/dL)	1.64 (1.04 to 2.97)	1.0 (0.8 to 1.0)	<0.001
NGAL (ng/mL)	459 (213 to 744)	120 (79 to 174)	<0.001
AOPP (*μ*mol/L)	505.1 (307.6 to 643.5)	115.7 (79.2 to 181.7)	<0.001
BNP (pg/mL)	409 (212 to 673)	135 (61 to 275)	<0.001
Sofa score	10 (8 to 12)	5 (4 to 5)	<0.001
Died (%)	32.1	16.7	0.08

NGAL: neutrophil gelatinase-associated lipocalin; AOPP: advanced oxidation protein products; BNP: brain natriuretic peptide; SOFA score: sequential organ failure assessment.

**Table 3:** Comparison of biochemical markers between AKI and No-AKI septic patients.

	AKI septic pts (*N* = 24)	No-AKI septic pts (*N* = 32)	*P *value
Male sex (%)	29.2	37.5	0.51
Age (years)	69 (50 to 71)	69 (45 to 76)	0.63
Creatinine (mg/dL)	2.3 (1.5 to 3.4)	1.2 (0.8 to 1.9)	0.0065
NGAL (ng/mL)	572 (308 to 819)	321 (154 to 573)	0.0425
AOPP (*μ*mol/L)	554.0 (366.8 to 717.6)	419.5 (286.8 to 607.4)	0.0425
BNP (pg/mL)	576 (291 to 1723)	348 (174 to 538)	0.0327
Sofa score	11 (8 to 13)	9 (7 to 12)	0.28
Died (%)	45.8	21.9	0.0575

NGAL: neutrophil gelatinase-associated lipocalin; AOPP: advanced oxidation protein products; BNP: brain natriuretic peptide; SOFA score: sequential organ failure assessment.

**Table 4:** Comparison of biochemical markers between AKI septic patients and AKI nonseptic patients.

	AKI septic pts (*N* = 24)	AKI nonseptic pts (*N* = 14)	*P* value
Male sex (%)	29.2	78.6	0.0033
Age (years)	69 (50 to 71)	76 (69 to 80)	0.0067
Creatinine (mg/dL)	2.3 (1.5 to 3.4)	1.0 (0.8 to 1.6)	<0.001
NGAL (ng/mL)	572 (308 to 819)	312 (141 to 633)	0.15
AOPP (*μ*mol/L)	554.0 (366.8 to 717.6)	118.9 (90.1 to 152.5)	<0.001
BNP (pg/mL)	576 (291 to 1723)	305 (134 to 559)	0.1055
Sofa score	11 (8 to 13)	5 (5 to 5)	<0.001
Died (%)	45.8	42.9	0.85

NGAL: neutrophil gelatinase-associated lipocalin; AOPP: advanced oxidation protein products; BNP: brain natriuretic peptide; SOFA score: sequential organ failure assessment.
